# Non‐Surgical Cosmetic Treatment Modalities for Interdental Papilla Reconstruction “Black Triangles”: An Evidence‐Based Review

**DOI:** 10.1111/jocd.70920

**Published:** 2026-05-13

**Authors:** Ayah Elhaj, Bashar Shatta, Sheila Nguyen, Ines Novo Pereira, Haidar Hassan

**Affiliations:** ^1^ Academic Plastic Surgery, Blizard Institute, Faculty of Medicine and Dentistry Queen Mary University of London London UK; ^2^ Faculty of Dental Medicine, University of Porto Porto Portugal; ^3^ Egas Moniz Center for Interdisciplinary Research (CiiEM), Egas Moniz School of Health & Science Caparica, Almada Portugal

**Keywords:** black triangles, cosmetic, deficiency, interdental papilla, non‐surgical, reconstruction

## Abstract

**Background:**

The interdental papilla plays a vital role in dental and facial aesthetics, as the loss of papilla manifests as unesthetic “black triangles”. In recent years, non‐surgical approaches such as hyaluronic acid injections, autologous platelet concentrates, laser therapy, and microneedling with vitamin C have emerged as alternatives to technically demanding and unpredictable surgical reconstruction.

**Aim:**

To evaluate the effectiveness, patient satisfaction, and safety of cosmetic non‐surgical treatment modalities for the correction of black triangles.

**Materials and Methods:**

A comprehensive literature search was conducted using the PubMed, Cochrane Library, Scopus, and Embase databases, from January 2013 to January 2024. All case series, cohort studies, and randomized controlled trials with relevant outcomes were included. The BestBETs methodology was used, and the risk of bias was assessed using the “Quality Assessment Tool for Quantitative Studies.”

**Results:**

A total of 19 human studies met the inclusion criteria. Of these, six studies were classified as level II evidence, while the remaining 13 were categorized as level III or level IV evidence. Based on level II evidence, hyaluronic acid injections were effective and safe for correcting the black triangles and improving the smile. Evidence on non‐surgical modalities such as autologous platelet concentrates, photobiomodulation therapy, and microneedling with vitamin C remains limited. Based on level II to level IV evidence, these conservative treatments are recommended as clinically effective for filling black triangle spaces, demonstrating high patient satisfaction and minimal adverse events.

**Conclusion:**

Non‐surgical cosmetic treatments offer a promising alternative for reconstructing interdental papilla deficiencies, with benefits including reduced complications and favorable aesthetic outcomes. However, the current evidence remains limited, and findings should be interpreted with caution. Further well‐designed, standardized clinical trials are required to establish the effectiveness, long‐term stability, and patient‐centred outcomes of these interventions.

## 1 Introduction

**FIGURE 1:**
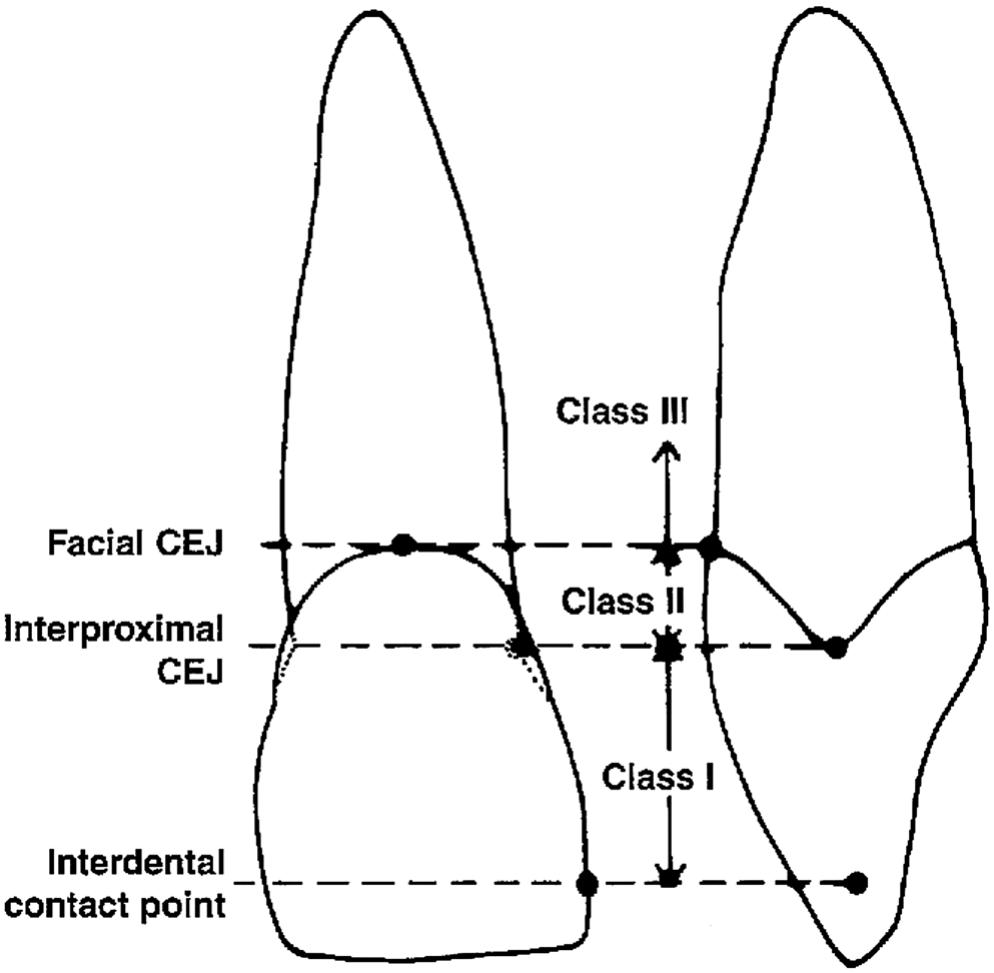
Nordland and Tarnow classification [5].

A beautiful smile is a key component of facial attractiveness and plays a vital role in psychological well‐being and self‐esteem. The demand for dental and medical cosmetic treatments to enhance smile aesthetics is increasing across orthodontics, plastic surgery, and periodontics [1, 2]. An attractive smile depends on the harmonious integration of hard and soft tissues, with the interdental papilla being essential for the balance between the “red” (gingiva) and “white” (teeth) components. The papilla fills the space between adjacent teeth, and its loss creates an open space referred to as a “black triangle” (BT), which compromises smile aesthetics, phonetics, and periodontal health. The etiology of papilla loss is multifactorial, including periodontal disease, orthodontic tooth movement, traumatic restorative procedures, and over‐contoured restorations [3, 4]. Nordland and Tarnow introduced the first and most widely used classification system for papilla defects as presented in (Figure 1) [5].

Al‐Zarea et al., in a narrative review of the literature, reported that approximately one‐third of individuals are affected by black triangles, citing figures suggesting a prevalence of 67% in adults aged 20 years and older compared with 18% in those under 20 years of age [6]. However, these estimates are not derived from primary population‐based epidemiological studies and should therefore be interpreted with caution [6]. According to Geld et al., patients ranked black triangles as the third most unattractive dental feature, following carious lesions and dark crown margins [7]. Consequently, the reconstruction of lost interdental papilla has become a priority in periodontal therapy, driven by increasing aesthetic demands. Treatment options are broadly classified into surgical and non‐surgical approaches. Surgical methods aim to restore both functional and aesthetic integrity of the papilla by recontouring and augmenting; however, they are highly technique‐sensitive, often unpredictable, and associated with uncertain long‐term stability [8]. These drawbacks have shifted attention toward non‐surgical modalities, with the potential to offer reduced complications, minimal downtime, and favorable aesthetic outcomes.

Several non‐surgical techniques have recently been investigated for papilla reconstruction. Hyaluronic acid (HA) injections have gained widespread use in medicine, cosmetics, and dentistry due to their desirable physicochemical properties, biocompatibility, and regenerative scaffold properties [9]. Similarly, autologous platelet concentrates, such as platelet‐rich plasma (PRP) and platelet‐rich fibrin (PRF), have been employed in periodontics to stimulate regeneration, accelerate wound healing, and enhance surgical results [10]. Additional non‐surgical options include liquid‐phase concentrated growth factor (LPCGF), which delivers bioactive molecules that promote angiogenesis and soft tissue healing; low‐level laser therapy (LLLT), which modulates cellular activity and enhances wound healing through photobiomodulation; and microneedling, which induces controlled micro‐injuries to stimulate neovascularisation and growth factor release [11, 12, 13].

Recent literature has examined both surgical and non‐surgical interventions for the reconstruction of interdental papilla deficiencies. The mini review by Barakat highlighted the potential of surgical and non‐surgical cosmetic strategies for papilla regeneration; however, it also noted persistent inconsistencies in protocols and outcome measures [14]. Similarly, Patel et al. systematically reviewed 45 studies and reported improvements across various modalities, including hyaluronic acid, platelet concentrates, and surgical grafting [15]. However, the authors emphasized that the evidence is inconsistent and constrained by non‐standardized outcome measures. Building on these insights, the present review critically appraises the clinical efficacy, patient satisfaction, and safety of current non‐surgical modalities for interdental papilla reconstruction.

## 2 Materials and Methods

An evidence‐based review was conducted using the BestBETs methodology, a structured, clinically oriented framework for appraising the best available evidence in response to a focused clinical question. This approach does not constitute a systematic review and does not adhere to formal systematic review methodology. To broaden the inclusiveness of the eligibility criteria and offer diverse perspectives to address the clinical question, a more permissive strategy was employed. However, our work adapted elements of systematic review techniques to comprehensively assess the existing evidence. The three‐part clinical question underpinning this review is shown in Table 1 [16].

**TABLE 1:** Three‐part research question.

Patient group	Healthy adults seeking conservative management of papilla loss
Intervention	Non‐surgical treatments, including hyaluronic acid, laser, growth factors, and microneedling
Relevant outcomes	Clinical efficacy (e.g., improvement in the black triangle area and measurement changes in papilla height), patient‐related outcomes, and safety

A comprehensive search was conducted in PubMed, the Cochrane Database, Scopus, and Embase from January 2013 up to September 2024, as outlined in Table 2. The search was limited to peer‐reviewed human studies published in English. Gray literature sources and clinical trial registries were not systematically searched.

**TABLE 2:** Electronic searches.

PubMed	A PubMed advanced search was done, including all the following terms “Effectiveness” AND “non‐invasive treatment” OR “non‐surgical” OR “cosmetic treatment” AND “black triangles” OR “dental papilla loss”
Cochrane Library and Embase	An Embase and Cochrane Library search with the terms “black triangles” AND “aesthetic treatment”
Scopus	A Scopus advanced search was done using the following terms “loss interdental papilla” OR “interdental papilla defect” OR “deficient papilla” OR “black triangle” AND “nonsurgical” OR “non invasive” OR “conservative treatment”

*Note:* KEY: The search strings presented above reflect the exact queries conducted during the literature search. It is acknowledged that the PubMed search strategy did not incorporate explicit Boolean operator grouping (parentheses), and that the search terms were primarily condition‐specific rather than intervention‐specific.

**FIGURE 2:**
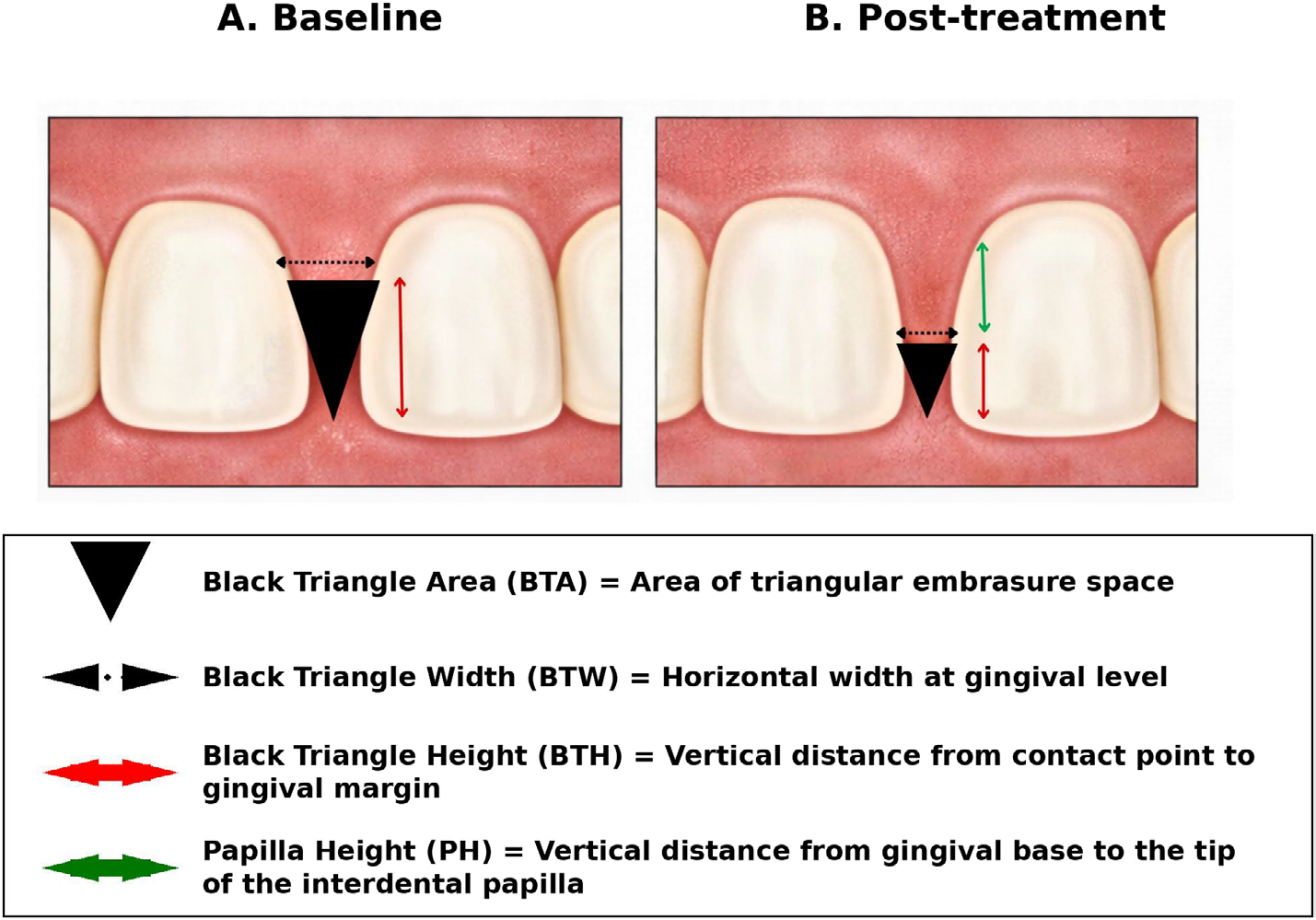
Schematic illustration showing black triangle area (BTA), black triangle height (BTH), and black triangle width (BTW) as commonly reported outcome measures at baseline (A) and post treatment using non‐surgical cosmetic interventions. The apex of the black triangle is directed coronally toward the interproximal contact point, while the base is located apically at the gingival level.

Two independent reviewers (AE and BS) have performed study selection and eligibility assessment, and data extraction based on the predefined inclusion and exclusion criteria shown in Table 3. Prior to formal screening, both reviewers jointly assessed a subset of studies to align interpretation of the eligibility criteria and ensure consistency in study selection. Any disagreements were resolved through discussion, and when consensus could not be reached, a third reviewer (HH) was consulted. Data extraction from human studies was performed independently by two reviewers (AE and BS). Extracted outcomes included changes in black triangle area (BTA), reported as percentage change and absolute differences before and after treatment, as well as patient‐reported outcome measures. In addition, black triangle height (BTH) and black triangle width (BTW) were recorded where available. These geometric outcome measures are illustrated schematically in (Figure 2) to aid clarity and consistency of interpretation across studies. The level of evidence for each included study was determined using the Oxford Centre for Evidence‐Based Medicine (2011) classification system [17]. The risk of bias was assessed using the “Quality Assessment Tool for Quantitative Studies” [18] developed by the Canadian public health sector to appraise methodological quality and provide evidence supporting public health research and interventions across a wide range of health‐related topics. Two reviewers (AE and BS) evaluated eight domains: selection bias, study design, confounders, blinding, data collection methods, withdrawals and dropouts, intervention integrity, and data analysis. Each domain was rated as “strong,” “moderate,” or “weak.” In accordance with the tool's guidelines, studies with no weak ratings were classified as strong overall; those with one weak rating as moderate; and those with two or more weak ratings as weak. Any differences of opinion were discussed with a third reviewer (HH).

**TABLE 3:** Inclusion and exclusion criteria for the studies included in the results.

Inclusion criteria	Exclusion criteria
Human studies with deficient interdental papilla	Animal studies
Studies reporting non‐surgical modalities	Studies reporting surgical treatments (sole treatment or combined with non‐surgical modalities)
English studies	All studies not written in the English language
	Reviews and expert opinion papers

## 3 Results

**FIGURE 3:**
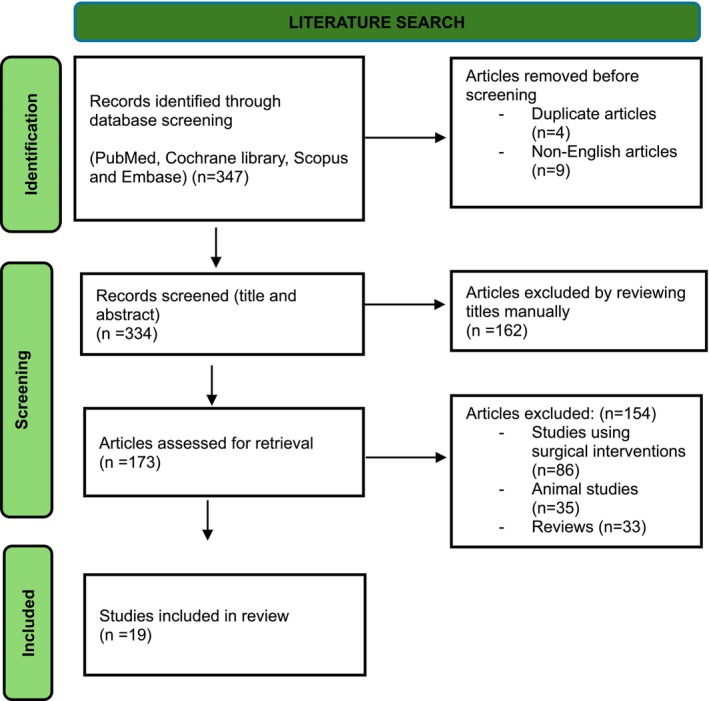
PRISMA flow chart [35] (updated 2020) represents the process of identifying, screening, and selecting studies for inclusion for this review.

A total of 19 articles were included in the final analysis [11, 12, 13, 19, 20, 21, 22, 23, 24, 25, 26, 27, 28, 29, 30, 31, 32, 33, 34]. The study selection processes are illustrated in The Preferred Reporting Items for Systematic reviews and Meta‐Analyses (PRISMA) flow diagram (Figure 3), included as a transparency tool to document study identification and selection [35]. Table 4 shows the characteristics of the 19 human studies that met the inclusion criteria based on the BestBETs protocol.

**TABLE 4:** Summary of literature review using non‐surgical modalities.

Aesthetic treatment modality	Author, date, and country	Study group and design	Study type (level of evidence)	Outcomes and key results	Study weakness
Hyaluronic Acid only (HA)	Ebrahimi et al. 2023, Iran [27]	4 healthy patients with a total of 20 sites of deficient papillae Nordland & Tarnow class I and II in the anterior area of the jaws 0.2 mL (1.6%) of HA gel was administered perpendicular to the injection sites. 2 injection sites on each papilla: one site 2–3 mm below the tip of the papilla and the other at the interdental tip Treatment sessions: total of 3 sessions with 2‐week intervals	Prospective clinical study, level III	3‐ and 6‐months follow‐ups with clinical measurement and picture analysis **Efficacy** Significant improvement *p* < 0.05 between the BTA, PH, and the distance between the apical point of contact to the tip of the papilla A significant difference in levels of BTA during 3 sessions *p* < 0.005 **Patient satisfaction** Not reported **Safety** No major adverse events	Small sample size and small number of areas examined Short follow‐up time Lack of three‐dimensional measurement to assess changes
	Ni et al. 2021, China [28]	24 healthy patients, 3 dropouts. 21 patients (2 male and 19 female) completed the study with 62 deficient papillae Nordland & Tarnow class I and II in the upper jaw 34 papillae were treated with hyaluronic acid (test group), 0.05–0.1 mL (16 mg/mL), and 34 papillae with saline solution injections (control group). Injections were given at the base of the deficient papilla Treatment sessions: multiple sessions repeated at 3 and 6 weeks	Randomized controlled trial, level II	6‐ and 12‐month follow‐up with Digital clinical photograph and clinical measurement **Efficacy** HA showed a significant reduction in BTA *p* = 0.004 HA showed significant improvement in PH *p* = 0.006 **Patient satisfaction** Not reported **Safety** No major adverse events	Predominantly female patients in this study Single‐centre study Lack of patient satisfaction with and perception of the procedure Lack of three‐dimensional measurement to assess changes
	Pitale et al. 2021, India [29]	7 healthy patients, 2 males and 5 females (mean age 30.96 years) with 25 papillae Nordland & Tarnow class I and II 0.2 mL (20 mg/mL) of Hyaluronic acid was administered 2–3 mm apical to the coronal tip of the papilla at a 45‐degree angle. Post‐treatment instructions were given to patients Treatment sessions: 1 session	Prospective clinical study, level IV	3‐ and 6‐month follow‐up with digital photographs and clinical measurements **Efficacy** Significant difference between contact point to gingival margin from baseline to 3‐ and 6‐months *p* = 0.01 At 3 months 52% sites showed complete papilla infill (13 out of 25 sites) **Patient satisfaction** Not reported **Safety** Mild discomfort at the injection site and irritation No major adverse events	Small sample size and short follow‐up periodLack of a control group
	Alhabashneh et al. 2021, Jordan [30]	27 patients (17 females, 10 males), mean age 37 years 21 patients (14 females and 7 males) completed the study with 86 papillae Nordland & Tarnow class I and II in the upper and lower jaw 0.2 mL of hyaluronic acid was administered at a 45‐degree angle with the bevel directed to the bone Treatment sessions total of 2 sessions with 3‐week intervals	Prospective clinical study, level III	3‐ and 6‐month follow‐ups by clinical measurement and digital photographs **Efficacy** Significant difference in the mean BTH with time *p* < 0.001 Patient satisfaction Some patients' feedback was reported, but no validated PROMs were used **Safety** Mild pain was reported by 3 patients for one week No major adverse events	Lack of randomized control Follow‐up limited to 6 months The concentration of the hyaluronic acid used wasn't specified
	Mandel et al. 2020, Hungary [33]	40 patients, 9 dropouts. 31 patients (23 females, 8 males) aged between 20 and 66 years old completed the study with 160 deficient papillae Nordland & Tarnow class I and II in the upper and lower jaw. Two different types of hyaluronic acids were used 1% Revident (test group *n* = 48 and untreated control *n* = 32) and 1% Flex Barrier (test group *n* = 50 and untreated control *n* = 30) Treatment sessions: 1 session	Randomized controlled trial, level II	1‐week and 1‐month follow‐ups by clinical measurement and picture analysis **Efficacy** Both HA gels reduced papilla defect vs. control Papilla defect size: At 1 month: Revident 86.1% (±22.6) Flex Barrier 96.0% (±8.8) Patient satisfaction Not clearly reported as validated PROMs **Safety** No major adverse events	Short follow‐up period up to 1 month. Only 4 patients of the Revident group were followed up for 18 months Lack of long‐term durability data The wide age range between participants may have affected the results Single injection protocol and single treatment session The outcomes of using two different materials weren't assessed by physical examination or by patient visual evaluation
	Patil et al. 2020, India [25]	Eight patients (three males, five females), with a mean age of 32 years. A total of 14 deficient papillae in the upper anterior area were included < 0.2 mL hyaluronic acid was administered 2–3 mm apical to the papilla tip Treatment sessions: up to 3 times, depending on whether a dark space is present, and if another injection was administered	Case series, level IV	3 months follow‐up by clinical measurement and picture analysis **Efficacy** Mean reduction in BTA and BTH BTH 0.85 ± 0.28 BTA 0.25 ± 0.12 Interdental papilla reconstruction rate 89.25% Complete infill in 8 sites out of 14 sites Partial infill on 6 sites out of 14 sites **Patient satisfaction** Not reported **Safety** No major adverse events	Small sample size and selections were limited to the deficient papilla in the upper anterior region Short follow‐up time up to 3 months Lack of a control group
	Singh and Vandana 2019, India [21]	10 healthy patients from both genders, 25–40 years. 35 deficient papillae were included, 17 in the upper jaw and 18 in the lower jaw < 0.2 mL different concentration of hyaluronic acid 1% (16 sites), 2% (7sites) and 5% (12sites). Injection was administered 2–3 mm apical to coronal tip of papilla Treatment sessions: total of 3 sessions with one‐week intervals	Prospective clinical study, level III	1‐, 3‐ and 6 months follow‐up by clinical and picture measurements **Efficacy** All HAs groups used showed enhancement. 5% showed greater effect 1% HA *p* = 0.004 2% HA *p* = 0.004 5% HA *p* = 0.001 **Patient satisfaction** Before treatment 62.5% slightly impressive smile 37.5% not impressive smile 25% BT extremely noticeable 75% BT slightly noticeable After treatment 12.5% extremely impressive smile 75% slightly impressive smile 12.5% not impressive smile **Safety** Pain at injection site was reported with HA 5% group Mild discomfort, swelling and tenderness lasted up to 1 week No major adverse events	Lack of control group Short follow‐up time up to 6 months
	Abdelraouf et al. 2019, Egypt [20]	10 healthy patients, 2 dropouts. A total 8 patients (2 males and 4 females, aged 21 to 47 years) with 30 deficient papillae Nordland & Tarnow class I and II anterior area. Patients were randomly divided into two groups: one (test group) received a hyaluronic acid injection. 0.1 mL (20 mg/mL) of hyaluronic acid was administered 2–3 mm apical to the tip of the papilla and with a 45‐degree angle. And second group received saline injections (control group) Treatment sessions: a total of 3 sessions every 3 weeks	Randomized controlled trial, level II	3‐ and 6 months follow‐up with picture analysis and clinical measurement **Efficacy** BTH decreased significantly after 3 months *p* < 0.001 BTA showed a significant difference after 3 months *p* < 0.001 BTA from baseline to 3 months 36.5% ± 24.4% and from baseline to 6 months 45.0% ± 28.5% Patient satisfaction At 6 months: HA reported a significant satisfaction with HA 45 ± 12.65 The satisfaction score after 6 months *p* = 0.002 **Safety** No major adverse events	Follow‐up is limited up to 6 months
	Bertl et al. 2017, Denmark [24]	22 healthy patients, 1 dropout. A total of 21 (12 females, 9 males) mean age 30 ± 6.4 years completed the study with 21 deficient papillae Patients were allocated into two groups: one group with saline injection (control group) and one group with hyaluronic acid. 1‐ creating a reservoir by injecting 0.18 mL immediately above the mucogingival junction, 2‐ 0.12 mL injection below the base of the deficient papilla, 3–0.06 mL injection 2–3 mm apical to the tip of the deficient papilla Treatment session: 2 sessions with 4‐week intervals	Randomized controlled trial, level II	3‐ and 6 months follow‐up by digital clinical photograph and clinical measurement **Efficacy** No significant change in BTH between the groups in BT over time. BTA measurements: Baseline 0.51 ± 0.31 After 3 months 0.47 ± 0.20 After 6 months 52 ± 0.26 **Patient satisfaction** Slight improvements were observed in both groups. VAS < 30 **Safety** Mild pain was reported after the injections *p* = 0.034 after 1st injection *p* = 0.064 after 2nd injection After 2nd injection, 2 patients reported sever pain and lip swelling	The HA used together with the injection technique resulted in a high rate of adverse events
	Lee et al. 2016, Korea [26]	13 healthy patients (6 males, 7 females) mean age 32 years. 57 deficient papillae in the upper front area One point injection technique used by inserting the injection 2‐3 mm below the interdental papilla with a 45‐degree angle with a total of 0.01 cc of hyaluronic acid used over 5 sessions Treatment sessions: 5 treatments with 3‐week intervals	Prospective study, level III	6 months follow‐up by clinical photograph analysis **Efficacy** 100% papilla reconstruction in 36 sites 19%–96% papilla infill in 21 sites Mean reduction in BTH 0.71 mm Mean reduction in BTA 0.20 mm^2^ **Patient satisfaction** Not reported **Safety** No major adverse events	Treated areas were restricted to the upper anterior area Difficulty in reproducing identical photo angles despite improved standardization Lack of control group Limited follow‐up period
	Awartani & Takakis 2016, KSA [22]	10 healthy patients, 1 dropout. 9 female patients (average age 36.4 years) with 17 deficient papillae Nordland & Tarnow class I and II in upper and lower jaws 0.2 mL of hyaluronic acid was injected 2‐3 mm apical to the tip of papilla. Post‐treatment instructions were given Treatment sessions: total 3 sessions treatments with 3‐week intervals	Case series, level IV	4‐ and 6 months follow‐up by clinical photographs **Efficacy** At 6 months 100% papilla infill in 3 sites 50% papilla infill in 8 sites Significant difference between baseline and 4‐ or 6‐months *p* < 0.0001 **Patient satisfaction** 2 of 9 patients were dissatisfied with the treatment. 5 out of 9 patients experienced post‐ treatment discomfort 66% of patients would choose to redo the treatment **Safety** pain at the injection site limited swelling at the injection site Transient tenderness	Small size and only females participated in the study Lack of a control group
Injectable Platelets Rich Fibrin (i‐PRF)	Puri et al. 2022, India [23]	4 patients with poor aesthetics with total of 6 deficient papilla in upper and lower jaw Approximately 1 mL of the liquid form of platelet rich fibrin was used after preparation. The injection was inserted 2‐3 mm apical to the papilla tip at a 45‐degree angle. Treatment session: 1 session	Case report, level IV	1‐, 3‐ and 6‐month follow‐up by clinical measurements, picture analysis and patients questionnaire **Efficacy** After 6 months: 100% filled papilla in 3 sites 75% filled papilla in 1 site 66.6% filled papilla in 2 sites **Patient satisfaction** At 6 months 50% (2 out of 4) BTs not noticeable at all and were extremely impressive with the results 50% (2 out of 4) BTs slightly noticeable and were slightly impressive with the results **Safety** Mild pain immediately after treatment	Small sample size with no specification of gender and age Lack of a control group. Short follow‐up period
Hyaluronic acid (HA) with and without Plasma Rich Growth Factors (PRGF)	Bal et al. 2023, Italy [19]	21 healthy patients, 2 dropouts. 19 patients (10 males, 9 females) aged 18‐45 years with 34 deficient papillae Nordland & Tarnow class I and II. Two groups: one group received 0.2 mL of 0.8% hyaluronic acid alone. One group received 0.2 mL of 0.8% hyaluronic acid followed by 0.2 mL plasma‐rich growth factors. Treatment sessions: 3 sessions with 3‐week intervals	Randomized controlled trial, level II	3‐, 6‐, and 12 weeks follow‐up by clinical and photographs measurements **Efficacy** Significant change with HA + PRGF at 6‐ and 12‐weeks *p* < 0.001 At 6 weeks mean change in BTA HA + PRGF 49.30% HA alone 20.24% At 12 weeks mean change in BTA HA + PRGF 77.42% HA alone 57.62% **Patient satisfaction** Not reported **Safety** No significant adverse events	Short follow‐up time up to 12 weeks The difference between groups was underestimated due to the mixed study design used Outcomes weren't assessed systemically
Albumin with platelet‐rich fibrin (Alb‐PRF) gel and hyaluronic acid gel (HA)	Vadiati Saberi et al. 2024, Iran [34]	10 healthy patients (6 males, 4 females) aged 20‐75 years with 46 deficient papillae Nordland & Tarnow class I and II. Two groups: one group received 0.2 mL of hyaluronic acid alone. One group received 0.2 mL of Alb‐PRF Treatment sessions: 2 sessions with 3‐week intervals	Randomized controlled trial, level II	3‐, and 6 months after first injection follow‐up by clinical and photographs measurements **Efficacy** BTA significantly reduced in Alb‐PRF and HA groups (*p* < 0.001) At 3 months mean change in BTA HA 56% Alb‐PRF 45% At 6 months mean change in BTA HA 64% Alb‐PRF 66% **Patient satisfaction** Higher in the HA group 65% than the Alb‐PRF group 60%, with no Statistically significant difference between groups (*p* = 0.219) **Safety** No significant adverse events	Small sample size, and the mean age between groups was significantly different Alb‐PRF group was older on average Short follow‐up period Study limitations weren't mentioned
Liquid phase concentrated growth factor injection (LPCGF) with low‐level laser therapy (LLLT)	Chen et al. 2022, China [12]	12 healthy females aged 18–60 years. 2 dropouts. 10 patients with 67 deficient papillae Nordland & Tarnow class I‐III in the upper or lower jaw 0.05–0.2 mL of LPCGF was injected at the base of the gingival papilla at a 45‐degree angle followed by Nd:YAG laser to the labial and lingual surfaces of the papilla Treatment sessions: Total of 6 injections at 2, 4, 8, 16 and 24 weeks after 1st treatment session. Laser was used once every 5 days until 14 days after the last treatment session	Prospective clinical study, level III	3‐, 6‐, and 12 months clinical measurements and picture analysis **Efficacy** Reduction in BTH from Baseline to 6 months: 0.46 ± 0.64 mm 12 months: 0.57 ± 0.63 mm. Reduction in BTA from Baseline to 6 months: 0.52 ± 0.92 mm^2^ 12 months: 0.58 ± 0.95 mm^2^ **Patient satisfaction** Not reported **Safety** No major adverse events reported	Small sample size, inclusion of females only Short follow‐up period Lack of patients reported outcomes Inconclusive LLLT parameters
Photobiomodulation therapy (PBMT)	Zanin et al. 2018, Brazil [13]	3 healthy patients (2 females and 1 male) aged 42–61 years with 9 deficient papillae PBMT with 660 nm diode laser into 2 stages: first stage total energy of 14 J before bleeding used for analgesic and simulation of microcirculation. Second stage: total energy of 14 J immediately after bleeding Treatment sessions: 2 sessions with 1‐week intervals	Case report, level IV	Twice a year follow‐up clinical picture. Follow‐up lasted 4–5 years **Efficacy** After 1 week: BTH reduced to 1 mm and base 0.5 mm After 2 weeks: complete papilla infill in all sites **Patient satisfaction** Favorable aesthetic outcomes **Safety** No major adverse events reported	Small sample size Lack of control group, risk of bias Study limitations weren't mentioned
	El Mobadder et al. 2024, Belgium [31]	34‐year‐old female with 1 deficient papilla between upper front teeth PBMT used with 635 nm diode laser at 4 points (coronal, apical, mesial and distal of papilla). Bleeding was initiated with a curette till blood filled the BT then PBMT used again for 50 s and an energy of 2.5 J per point. Post‐treatment instructions were given Treatment sessions: total of 3 sessions, repeated 5 and 10 days after the first session	Case report, level IV	3‐months follow‐up by clinical photographs **Efficacy** Slight increase of PH Reduction in BT appearance **Patient satisfaction** Not reported **Safety** No major adverse events reported	Very small sample size and short follow‐up time. Lack of clinical measurements to validate the observations of the study Lack of patients reported outcomes and side effects
Tissue‐Engineering technology (TEP) includes bone marrow mesenchymal cells (BMMSCs), platelet rich plasma (PRP) and hyaluronic acid (HA)	Yamada et al. 2013, Japan [32]	10 healthy patients, 7 females and 3 males aged 20‐64 with 11 deficient papillae Injectable TEP prepared by mixing BMMSCs with PRP and HA mixture and thrombin mixture The mean volume injected was 1.32 ± 0.25 mL into the aspect next to the papilla. Treatment sessions: up‐to 5 sessions	Prospective Clinical trial, level III	Mean follow‐up period 55.3 ± 17.7 months by clinical measurements and picture analysis **Efficacy** Significant improvement BTs mean values 2.55 ± 0.89 mm **Patient satisfaction** High patient satisfaction **Safety** No sever adverse effects reported	Small sample size The injection technique and angulation weren't clear. In addition to the interval time between the treatment sessions. Experimental techniques and complex
Vitamin C injection with microneedling	Ahuja et al. 2022, India [11]	15 healthy patients aged 25‐35 years with15 deficient papillae Nordland & Tarnow class I and II Single point technique was used; 1‐1.5 mL of Vitamin C injection was injected 2‐3 mm apical to the papilla at 45‐degree angle. Followed by microneedling at the required area. Treatment sessions: 6 sessions with 7‐days intervals	Prospective study, level III	Follow‐up 7 days after the 6th sessions by clinical measurement and picture analysis. **Efficacy** A significant variation in the mean Of PH over time *p* = 0.002 The mean increase in PH from baseline to third follow‐up 3.20 ± 0.274 mm, 4.10 ± 0.652 mm, respectively **Patient satisfaction** Not reported **Safety** Minimal transient discomfort No sever adverse effects reported	Small sample size, very short follow up time

Various non‐surgical cosmetic approaches for restoring BTs were identified, including HA injections, injectable growth factors, laser therapy, microneedling, and vitamin C injections. Notably, no studies providing level I evidence were identified during the search. Of the included studies, six were classified as level II evidence [19, 20, 24, 28, 33, 34]. Four of these studies used HA as a standalone treatment [20, 24, 28, 33], while two studies combined it with plasma‐rich growth factors [19, 34]. The remaining 13 studies were classified as level III or level IV evidence, evaluating interventions such as HA, laser therapy, microneedling, and injectable growth factors [11, 12, 13, 19, 21, 22, 23, 25, 26, 27, 29, 30, 31, 32]. Level V evidence, including reviews and expert opinion papers, was excluded as outlined in the exclusion criteria.

Regarding methodological quality, all 19 included studies demonstrated at least one domain of concern. Fourteen studies were assessed as high risk of bias [11, 12, 13, 19, 21, 22, 23, 25, 26, 27, 29, 30, 31, 32, 34], and five studies involving HA interventions were rated as moderate risk [19, 20, 24, 28, 33]. Notably, no study was classified as low risk of bias. These findings limit the certainty of the available evidence. A detailed summary of the risk‐of‐bias assessment is provided in Table 5 [18].

**TABLE 5:** Risk of bias.

Authors and publication Year	Selection bias	Study design	ConFounders	Blinding	Data collection method/s	Withdrawals and dropouts	Overall global rating
Ebrahimi et al. (2023) [27]	Moderate	High	High	High	Moderate	Not applicable	High
Ni et al. (2021) [28]	Low	Low	Moderate	Moderate	High	Moderate	Moderate
Pitale et al. (2021) [29]	Moderate	High	High	High	Moderate	Not applicable	High
Alhabashneh et al. (2021) [30]	Moderate	High	High	High	Moderate	Not applicable	High
Mandel et al. (2021) [33]	Low	Low	Moderate	Moderate	High	Moderate	Moderate
Patil et al. (2020) [25]	Moderate	High	High	High	Moderate	Not applicable	High
Singh & Vandana (2019) [21]	Moderate	High	High	High	Moderate	Not applicable	High
Abdelraouf et al. (2019) [20]	Low	Low	Moderate	Moderate	High	Moderate	Moderate
Bertl et al. (2017) [24]	Low	Low	Moderate	Moderate	High	Moderate	Moderate
Lee et al. (2016) [26]	Moderate	High	High	High	Moderate	Not applicable	High
Awartani& Takakis (2016) [22]	Moderate	High	High	High	Moderate	Not applicable	High
Bal et al. (2023) [19]	Low	Low	Moderate	Moderate	High	Moderate	Moderate
Vadiati Saberi et al. (2024) [34]	Moderate	Low	Moderate	High	Moderate	Not applicable	High
Puri et al. (2022) [23]	Moderate	High	High	High	Moderate	Not applicable	High
Zanin et al. (2018) [13]	High	High	High	High	Moderate	Not applicable	High
El Mobadder et al. (2024) [31]	High	High	High	High	Moderate	Not applicable	High
Chen et al. (2022) [12]	Moderate	High	High	High	Moderate	Not applicable	High
Yamada et al. (2013) [32]	Moderate	High	High	High	Moderate	Not applicable	High
Ahuja et al. (2022) [11]	Moderate	High	High	High	Moderate	Not applicable	High

Table 6 summarizes reported outcomes related to complete and partial papillary fill across included studies. However, due to heterogeneity in study design, outcome definitions, and assessment methods, these data are presented for descriptive purposes only and should not be interpreted as directly comparable.

**TABLE 6:** Descriptive summary of reported papillary fill outcomes across included studies (non‐comparable data).

Author and publication, year	Treatment modality	No. of sites	No. of sites with complete infill	No. of sites with partial infill	% Of papillary fill	Number of applications	Interval time between applications
Ebrahimi et al. (2023) [27]	HA	20		20	70.256	3	2 weeks
Ni et al. (2021) [28]	HA	68			NR	3	3 weeks
Pitale et al. (2021) [29]	HA	25	12	13	48–100	1	NR
Alhabashneh et al. (2021) [30]	HA	86			NR	2	3 weeks
Mandel et al. (2021) [33]	HA	160		160	86.1–96.0	1	NR
Patil et al. (2020) [25]	HA	14	8	6	16–100	3	3 weeks
Singh & Vandana (2019) [21]	HA	42	1	41	50–100	3	1 week
Abdelraouf et al. (2019) [20]	HA	16		16	NR	3	3 weeks
Bertl et al. (2017) [24]	HA	11		11	NR	2	4 weeks
Lee et al. (2016) [26]	HA	43	29	14	19–100	Up to 5	3 weeks
Awartani& Takakis (2016) [22]	HA	17	3	8	2.1–100	3	3 weeks
Bal et al. (2023) [19]	HA+ PRGF	18		18	42.28–91.19	3	3 weeks
Vadiati Saberi et al. (2024) [34]	HA + Alb‐PRF	46		46	56–64	2	3 weeks
Puri et al. (2022) [23]	I‐PRF	6	3	3	66.6–100	1	NR
Zanin et al. (2018) [13]	PBMT	9	9		100	2	1 week
El Mobadder et al. (2024) [31]	PBMT	1					
Chen et al. (2022) [12]	LPCGF with LLLT	67	24	43	NR	6	2–8 weeks
Yamada et al. (2013) [32]	TEP	11		11	NR	Mean 2.2 ± 1.6	NR
Ahuja et al. (2022) [11]	Vitamin C injection and Microneedling	15		15	NR	6	1 week

In short, the included studies consistently demonstrated positive results with HA injections as a non‐surgical approach for reconstructing deficient interdental papillae. Despite using different commercial HA formulations, all evaluated types showed consistent clinical efficacy, yielding comparable positive results. Evidence from five randomized controlled studies (RCTs) supports these findings [19, 20, 24, 28, 34]. Significant improvements were reported over a follow‐up period of 6‐12 months, a mean increase in papilla height (PH) of 0.28 mm, and reductions in black triangle area (BTA) of up to 0.45mm^2^ in HA‐treated groups compared to saline controls [28]. A clear concentration response relationship was observed, as higher HA concentrations were associated with superior clinical outcomes; in particular, 5% HA achieved more significant improvements in PH and BT dimensions compared with lower concentration, including 1% and 2% HA [22]. Lower‐concentration formulations demonstrated more modest effects and were not directly comparable to higher‐concentration protocols. Collectively, these findings indicate that clinical outcomes following HA injection are strongly influenced by concentration and should be interpreted within the context of the specific formulation used.

In studies evaluating adjunctive or alternative biologic approaches, combination therapies and regenerative modalities demonstrated favorable clinical outcomes. In a RCT, Bal et al. reported that HA combined with plasma‐rich growth factors resulted in a mean reduction in deficient papillary area of 77.42% at 12 weeks, compared with 57.62% for HA alone [19]. In a separate RCT, Vadiati Saberi et al. reported greater reductions in BTA and higher patient satisfaction scores with albumin‐enriched PRF compared with HA monotherapy at 6 months [34]. Alternative modalities were less commonly studied than HA interventions. In the case series by Puri et al., the use of injectable platelet‐rich fibrin (i‐PRF) resulted in complete papilla infill in 3 sites, 75% infill in 1 site, and 66.6% infill in 2 sites accompanied by high levels of patient‐reported satisfaction [23]. In a prospective study, Chen et al. demonstrated reductions in BTA (0.58 ± 0.95 mm^2^) and BTH (0.57 ± 0.63 mm) at 12 months following treatment with LPCGF combined with LLLT [12]. In parallel, Zanin et al. reported that hemolaser therapy achieved complete interdental papilla fill in all treated sites, with stability maintained over a long‐term follow‐up of 4‐5 years [13].

Microneedling combined with vitamin C injections was evaluated in a single prospective study by Ahuja et al., which reported a mean increase in PH of 0.900 ± 0.418 mm at long term follow up and 0.340 ± 0.152 mm at short term follow up [11]. In a separate prospective study, Yamada and authors employed a tissue‐engineered injectable papilla using bone marrow‐derived mesenchymal stem cells combined with PRP and HA, which resulted in significant improvements in BT reduction, with a mean PH increase of 2.55 ± 0.89 mm maintained over an extended follow‐up period of up to 69 months [32].

Only seven studies assessed patient satisfaction following treatment [20, 21, 22, 23, 30, 33, 34]. Validated patient‐reported outcome measures (PROMs) were not employed in most of these studies; only one RCT classified as level II evidence, used a visual analogue scale (VAS) to evaluate patient satisfaction [20, 34]. Most satisfaction data were derived from studies using HA interventions, with a few reports involving i‐PRF and tissue‐engineered papilla. Although reported satisfaction was generally positive, the lack of standardized assessment tools limits the reliability and comparability of these findings. Furthermore, most studies reporting on patient satisfaction were level III‐IV evidence, highlighting the need for future RCTs to incorporate standardized, validated PROMs to better assess patient‐centred outcomes.

The safety profile of non‐invasive modalities was consistently favorable across the included studies, with no major complications reported. The most common adverse events were mild, transient discomfort, swelling, or tenderness at the injection site [11, 21, 22, 24, 29, 30]. These were primarily associated with HA injections and typically resolved within a few days. PRF and platelet‐rich growth factors injections were also well tolerated, with no adverse events reported [19, 23]. Rare complications, such as localized granuloma formation and transient lip swelling, were reported in an isolated case involving HA, as described by Bertl et al., these resolved without long‐term sequelae [36].

## 4 Discussion

The findings of the present review are broadly consistent with, yet extend beyond, those reported by Barakat [14] and Patel et al. [15] who highlighted the potential of non‐surgical approaches for interdental papilla reconstruction. The present review extends this work by focusing on non‐surgical cosmetic modalities and providing a structured appraisal of the available evidence across treatment efficacy, safety, and patient‐reported outcomes. However, it must be acknowledged that the evidence in these latter domains remains limited, with studies inconsistently reporting safety data and rarely employing validated PROMs. Across the included studies, individually reported improvements in PH ranged descriptively from approximately 0.3 mm to 2.55 mm, and reductions in BTA of up to 0.45 mm^2^ were reported within individual RCTs. These values are presented for descriptive and contextual purposes only, given that no formal data pooling was performed owing to considerable heterogeneity in study designs, outcome measurement methods, treatment protocols, and follow‐up durations. Also, it is important to emphasize that the overall body of evidence is limited in methodological strength; no Level I evidence was identified, six studies were classified as Level II, and the remaining 13 were Level III or IV. Furthermore, all included studies were judged to have at least one methodological concern, with 14 studies rated as high risk of bias, five as moderate risk, and no study was rated as low risk of bias. Accordingly, all findings must therefore be interpreted with caution.

Overall, the available evidence suggests that non‐surgical cosmetic interventions may be associated with favorable aesthetic outcomes. Prospective studies reported partial or complete papilla fill, with some studies documenting complete correction at treated sites [12, 13, 21, 22, 23, 24, 25, 26]. However, treatment protocols varied considerably with respect to the number of applications, injection volumes, and intervals between sessions, underscoring a lack of standardization across studies. In addition, clinical outcomes are influenced by anatomical factors beyond the treatment modality itself, including bone crest to contact point distance (BC‐CP), interproximal width, gingival tissue thickness, tooth form, and axial root inclination [37]. For example, BC‐CP distance of 5 mm or less is generally associated with complete papilla fill, whereas distances exceeding 6–7 mm are more likely to result in persistent deficiencies. Most included studies did not systematically account for these parameters, which may partly explain the variability in reported outcomes. Future studies should incorporate standardized anatomical characterization to enable more meaningful interpretation of treatment effects.

Among the evaluated modalities, HA injections were the most extensively investigated modality and were associated with consistently reported positive outcomes within the included studies, although these findings are derived predominantly from studies with moderate to high risk of bias. RCTs reported significant improvements in PH and reductions in BTA over 6–12 months, with higher concentrations producing superior results [20, 24, 28, 33, 34]. However, the clinical effect of HA appears to be transient, often necessitating repeated injections, and outcomes may vary between commercially available formulations. Variability in HA concentration and treatment frequency represents an important source of heterogeneity among studies. While higher concentrations and repeated treatment sessions are biologically and clinically expected to enhance papilla fill, the current evidence does not allow reliable dose‐ or frequency‐specific conclusions. Despite these encouraging results, the additional cost and increased procedural complexity associated with repeated treatments may restrict widespread clinical application. Importantly, there is a marked imbalance in the available evidence across modalities: hyaluronic acid was evaluated in 14 of the 19 included studies, counting five RCTs, and is therefore the only modality for which tentative conclusions regarding effectiveness can be drawn.

All other modalities are supported by limited data and should be considered preliminary. Moreover, for alternative biologic and device‐based modalities, the available evidence is derived from small case series, single prospective studies, or heterogeneous protocols. Accordingly, findings related to these approaches should be interpreted cautiously in light of the limited evidence. For example, i‐PRF was reported to achieve complete or partial papilla infill with high patient satisfaction, although current evidence is limited to small case series, reducing generalisability [32]. Similarly, microneedling with vitamin C demonstrated a measurable increase in PH; however, evidence is limited to a single study and should be interpreted as preliminary, with long‐term stability remaining uncertain [11]. Extending beyond these biologic methods, tissue‐engineered strategies such as mesenchymal stem cells combined with HA or PRP yielded the most substantial long‐term improvements, with PH gains sustained over extended follow‐up periods [32]. Despite their potential, these advanced regenerative approaches remain experimental, requiring complex preparation, ethical approval, and high costs, which currently limit their clinical feasibility. Moving from biologic to device‐based interventions, laser‐assisted techniques, including LLLT and hemolaser therapy, have also demonstrated favorable reductions in BT dimensions and, in some cases, complete papilla fill maintained over several years [12, 13, 31]. Nevertheless, the high cost of equipment, the need for specialized training, and protocol variability restrict broader application.

The interpretation of findings from this review is constrained by two key factors: study quality and outcome heterogeneity. All included studies exhibited at least one methodological limitation, with the majority classified as high risk of bias due to small sample sizes, lack of blinding, and short follow‐up durations. In addition, outcome measures varied considerably across studies, including BTA, BTH, PH, percentage papillary fill, and subjective or photographic assessments, often without standardized or validated measurement protocols. In particular, only a small number of studies evaluated patient satisfaction, and validated PROMs were rarely used. Given that aesthetic outcomes and patient satisfaction are central to the management of interdental papilla deficiencies, the lack of standardized and validated PROMs instruments limits meaningful interpretation of treatment success. Future studies should prioritize the use of validated and standardized PROMs to improve comparability and strengthen patient‐centered outcome assessment. As a result, the overall conclusions of this review are necessarily qualitative and descriptive. Although the consistent direction of findings provides a degree of cautious optimism, non‐surgical cosmetic interventions should currently be regarded as adjunctive rather than definitive treatment options. Future research should prioritize well‐designed, adequately powered RCTs with standardized outcome measures, validated patient‐reported outcomes, and longer follow‐up periods. The development of a core outcome set for interdental papilla reconstruction would further enhance comparability and strengthen the evidence in this field.

### 4.1 Limitations

As outlined in the methodology section, this review does not constitute a systematic review; no protocol was prospectively registered, the search strategy was not exhaustive, and the methodological safeguards required for systematic reviews were not applied. It should therefore be interpreted within the methodological context of the BestBETs framework, which provides a practical, clinically oriented approach to evidence synthesis for focused clinical questions.

In addition to these methodological considerations, several limitations of the included studies must also be considered. First, the number of studies evaluating non‐surgical cosmetic interventions for interdental papilla reconstruction remains limited, and most available evidence originates from small‐scale case series or non‐randomized clinical trials. Consequently, the overall strength of evidence is moderate to low, and the potential for publication bias cannot be excluded. Also, the search strategy was restricted to English‐language publications, and gray literature and clinical trials registries were not systematically searched. Moreover, the search terms were primarily condition‐specific rather than intervention‐specific. This means that terms such as ‘injectable PRF,’ ‘platelet‐rich fibrin,’ ‘photobiomodulation,’ and ‘low‐level laser therapy’ were not systematically included, which may have reduced the sensitivity and comprehensiveness of the search, particularly for emerging modalities. Second, considerable heterogeneity was observed across studies in terms of injection techniques, treatment frequency, follow‐up duration and outcome assessment methods, precluding quantitative meta‐analysis. The short duration of effect of HA, the need for repeated injections, and variability among formulations, concentrations, and treatment frequency further limit the strength of the evidence. Although higher concentrations and multiple sessions are expected to enhance papilla fill, current data do not allow dose‐ or frequency‐specific conclusions, highlighting the need for standardized protocols in future studies. Third, most studies have focused on the treatment of deficient papillae Nordland & Tarnow class I and II in the upper and lower jaws, predominantly in female patients, which can limit the generalisability of the results. In addition, validated PROMs were rarely employed, and safety data were inconsistently reported, limiting the ability to draw firm conclusions regarding patient satisfaction and long‐term treatment safety.

A further limitation relates to outcome assessment methodology. Many studies relied on two‐dimensional photographic analysis, which is susceptible to variability in image acquisition, including angulation, magnification, and lighting conditions, thereby reducing reproducibility. Emerging three‐dimensional digital technologies, such as intraoral scanning and structured light imaging, may offer more accurate and standardized assessment of papilla dimensions and should be incorporated into future research to mitigate potential measurement bias.

Finally, the review focused on cosmetic non‐surgical modalities and direct comparisons with surgical techniques were beyond its scope.

### 4.2 Clinical Recommendations

The following recommendations are provisional and intended to guide clinical decision‐making in the absence of stronger evidence. To note that no included study was rated as low risk of bias, and no Level I evidence was identified. Accordingly, these recommendations should not be interpreted as definitive practice guidelines and remain subject to revision as higher‐quality evidence becomes available.

That said, within the limitations of available evidence, clinicians should approach non‐surgical cosmetic management of interdental papilla deficiencies with careful case selection and realistic patient expectations. HA fillers represent the most extensively studied non‐surgical modality and have demonstrated consistently reported positive outcomes and a favorable short‐term safety profile, particularly for Nordland & Tarnow class I and II papillary deficiencies in both jaws. Autologous platelet concentrates may offer additional regenerative potential, particularly when combined with HA, though evidence remains preliminary. Other emerging modalities, including photobiomodulation, microneedling, and vitamin C adjunctive therapy, show early promise but require further validation before routine clinical use.

Clinicians should prioritize individualized treatment planning and informed consent that reflects the experimental nature of many of these techniques. Documentation of both clinical and patient‐reported outcomes, along with systematic safety monitoring, is strongly encouraged to strengthen future evidence. Until higher‐quality evidence becomes available, these interventions should be considered adjunctive rather than definitive alternatives to established surgical methods.

## 5 Conclusion and Future Perspective

Available evidence suggests that non‐surgical cosmetic interventions may offer a potential approach for the management of interdental papilla deficiencies. HA injections have captured most of the attention in clinical research. However, the overall body of evidence remains limited in methodological strength, with no level I evidence, small sample sizes, and a predominance of studies at moderate‐to‐high risk of bias. As for the effects of other modalities, including autologous platelet concentrates, photobiomodulation therapy, microneedling, and tissue‐engineered approaches, data is currently limited and should be considered preliminary. In addition, heterogeneity in outcome measures and variability in treatment protocols restrict direct comparison between studies and limit the strength of overall conclusions.

While reported clinical and aesthetic improvements are encouraging, the long‐term stability of these interventions remains uncertain, and patient‐reported outcomes are inconsistently assessed. Therefore, current findings should be interpreted with caution. Future research should focus on well‐designed, adequately powered RCTs with standardized outcome measures, longer follow‐up periods, and the incorporation of validated PROMs to better inform clinical practice.

## Author Contributions

All authors contributed to the study conception and design. Acquisition of data, analysis and interpretation of data were performed by (AE, BS). The first draft of the manuscript was written by (AE, BS), and all authors commented on previous versions of the manuscript and provided critical revisions. All authors read and approved the final manuscript.

## Funding

The authors have nothing to report.

## Ethics Statement

The authors confirm that the ethical policies of the journal, as noted on the journal's author guidelines page, have been adhered to. No ethical approval was required as no original research data was presented. There are no human participants in this article, and informed consent is not required.

## Conflicts of Interest

The authors declare no conflicts of interest.

## Data Availability

The data that support the findings of this study are available from the corresponding author upon reasonable request.
